# Training in cardiothoracic transplantation in the 21st century: Inspired apprenticeships with a scientific trend

**DOI:** 10.21542/gcsp.2024.27

**Published:** 2024-08-01

**Authors:** Espeed Khoshbin

**Affiliations:** 1 Consultant in Cardiac Surgery Transplantation and Mechanical Circulatory Support, Cardiac Training Lead, Royal Brompton and Harefield Hospital as part of Guys and St Thomas NHS Trust, London, UK; 2 National Lead for Transplant Education (Society of Cardiothoracic Surgeons of Great Britain and Ireland); 3 Honorary Clinicals Senior Lecturer, National Heart and Lung Institute, Imperial College London, UK

Sir Peter Medawar was a 20th century British biologist and Nobel prize winner. He was a pioneer in the field of tissue rejection and transplantation. He inspired many, including Christian Barnard and Sir Magdi Yacoub, surgeons who need no introduction to the reader (Figure 1).

As a passionate medical student from the United Kingdom who was inspired by great transplant surgeons, the author was encouraged by his mentor to gain experience in transplantation by visiting the United States (Figure 2). What followed in his short apprenticeship just before the turn of the century was the recognition of the need for a robust, structured, and scientific training in the field of cardiothoracic transplantation for surgeons.

Recently we have witnessed technological advances such as tissue engineering and gene modification, however many recent advances continue to require surgical interventions. An example of this is xenotransplantation. It is therefore essential that transplant surgeons are trained not only in surgical skills but also in transplantation at a molecular level. A purely clinical model of surgical training is no longer practical.

In the UK, The Royal College of Surgeons (RCS) and the Joint Committee on Intercollegiate Examinations (JCIE) are responsible for training in cardiothoracic surgery. In 2009 the National Health Service (NHS) and Speciality Advisory Committee (SAC) in cardiothoracic surgery, jointly reviewed and recognized that the number of transplant surgeons trained in the UK was insufficient to meet demand. Consequently, a national fellowship was established with an aim to deliver transplant-specific surgical training and to supply competent surgeons needed to sustain the UKs transplant programme. The fellowship was subsequently audited in 2017 and proved to be successful in training competent surgeons. Recommendations were made to the SAC to improve the quality of training. However, the number of interested trainees remains scarce.

The lack of qualified transplant surgeons remains a global problem. The highly demanding and unsociable lifestyle of a transplant surgeon makes this career unattractive to all but a few inspired individuals. Therefore, a holistic, multi-faceted solution is required to train transplant surgeons of the future.

**Figure 1.**
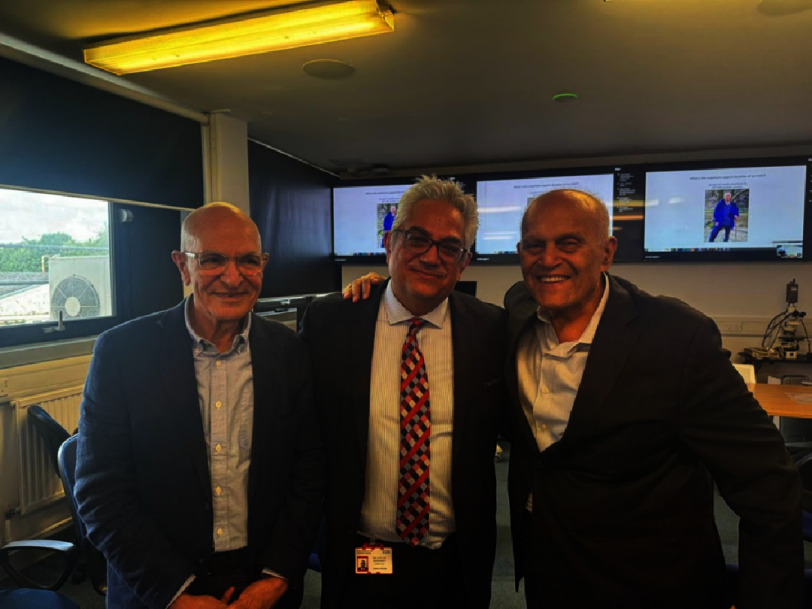
Picture taken at the Harefield transplant training course in May 2024, held at the Magdi Yacoub institute in London. The course is endorsed by the Society of Cardiothoracic Surgeons of Great Britain & Ireland and certified by the Royal College of Surgeons of Edinburgh. From right to left, Transplant surgeons: Asghar Khaghani, Espeed Khoshbin and Magdi Yacoub.

**Figure 2.**
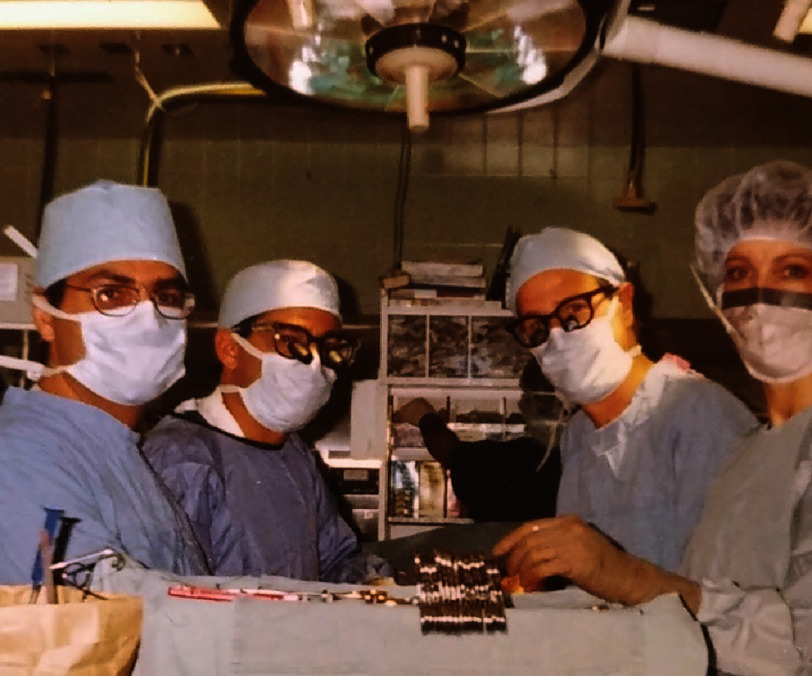
Espeed Khoshbin during his student elective with Bartley P Griffith at Pittsburgh Presbyterian Hospital in 1996.

**Figure 3.**
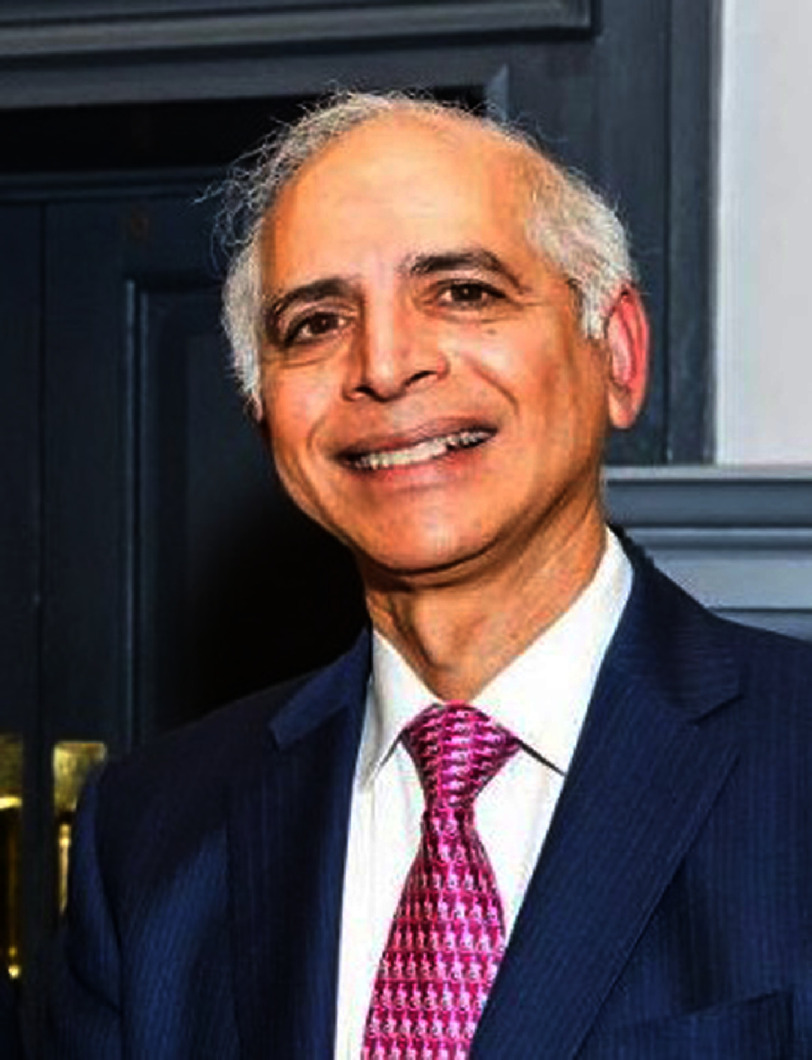
Si Prasad: early surgical career mentor to Espeed Khoshbin. Picture taken at the trainers and trainees event in July 2023.

## Inspiring the young generation

Being inspired to become a transplant surgeon in the early stage of a surgeon’s career would plant a strong foundation based on curiosity, interest, and emotional engagement (Figure 3). This early engagement is essential for an intensive life dedicated to patient care in all aspects of medicine.

## An international approach

The International Society of Heart and Lung Transplantation (ISHLT) has formed a global transplant community. This has facilitated access to international scholarships and awards for young surgeons.

## Certification

Undergraduate and post graduate certification by universities and surgical bodies would ensure career progression as part of continuous professional development (CPD).

## Formal training courses

**Figure 4.**
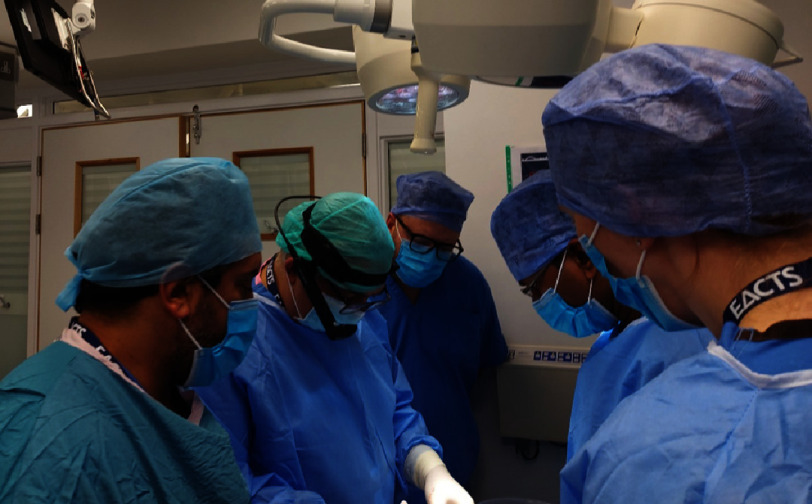
European Association of Cardiothoracic Surgery (EACTS) advanced transplant surgery course at Freeman Hospital, United Kingdom. Steve Clark and Espeed Khoshbin utilizing artificial intelligence during a cadaveric demonstration.

As the landscape in surgical practice changes to a complex interplay between science and surgery, it is essential for the surgical training to follow the trend from a predominantly apprenticeship approach to a more scientific, evidence based multidisciplinary model. It is essential for surgeons to have an in-depth understanding of the current complex nature of transplantation. Courses should therefore facilitate scientific lectures and group discussions as well as hands-on cadaveric wet labs (Figure 4).

## Research in education

To make surgical training in transplantation more effective there is a need to invest in education research. The use of artificial intelligence to train surgeons outside the operating room is a concept known as *training an expert novice*. This will also facilitate the process of skill assessment and maintaining training standards at an early stage in surgical training.

## Mentorship programmes

It is important to recognise that, without mentorship, surgeons will not reach the level of competency required to conduct a complex transplant procedure. For the more advanced surgeons formulating a structured surgical mentorship programme with measurable outcomes is essential. This will ensure effective use of training time comparable to training in other surgical super specialities.

## Surgical research

It is important for the future surgeons to have an in-depth understanding of research methodology. As surgical practice becomes more scientific there will be a need for collaboration between surgeons, and pure scientists.

## Funding

End-stage heart and lung diseases continue to be a significant cause of mortality and morbidity. Training competent transplant surgeons requires investment. It is likely that in the foreseeable future there will remain a need for transplant surgeons. Therefore, training transplant surgeons is an investment for the future of humanity.

## Reaching out

There remains our obligation to reach out to the less fortunate societies where transplant infrastructure is lacking. It is impossible to aspire to treat end-stage heart and lung disease worldwide without innovation and training in innovative practices.

## Conclusion

Surgical training in cardiothoracic transplantation is evolving. Training competent, innovative, and scientifically-minded transplant surgeons requires investment in a structured, multifaceted training programme. Early recruitment and retention through mentorship and appropriate funding is the key to training competent surgeons. Further research in surgical education is essential.

